# Supplementary material for: MetaRibo-Seq measures translation in microbiomes
Source: Nat Commun. 2020 Jun 29;11:3268. doi: 10.1038/s41467-020-17081-z (PMC7324362; doi:10.1038/s41467-020-17081-z)
Supplement: Supplementary file 10 — Supplementary Data 7 [file 41467_2020_17081_MOESM10_ESM.zip › File2/Confidence_High_Taxonomy/83609_out.krona.html]

Javascript must be enabled to view this page.

members
magnitude
magnitudeUnassigned
count
unassigned
taxon
rank

83609\_out

130

2
130
superkingdom

976
129
phylum

class
129
200643

171549
129
order

815
family
129

31

SRS014683\_contig\_number\_18300SRS015579\_contig\_number\_26400SRS016381\_contig\_number\_contig-100\_863.864SRS016495\_contig\_number\_contig-100\_2023.127273SRS017307\_contig\_number\_contig-100\_1922.183462SRS018836\_contig\_number\_contig-100\_220.163301SRS021948\_contig\_number\_contig-100\_956.202205SRS022609\_contig\_number\_contig-100\_3368.259064SRS042966\_contig\_number\_13368SRS045004\_contig\_number\_5714SRS050801\_contig\_number\_3390SRS053398\_contig\_number\_12059SRS054059\_contig\_number\_contig-100\_276.35629SRS054905\_contig\_number\_14870SRS063813\_contig\_number\_contig-100\_8000.8001SRS075963\_contig\_number\_contig-100\_105.105SRS077730\_contig\_number\_contig-100\_3677.110703SRS098620\_contig\_number\_contig-100\_48435.48435SRS101441\_contig\_number\_contig-100\_553.553SRS103987\_contig\_number\_contig-100\_2335.200733SRS1041139\_contig\_number\_4792SRS142923\_contig\_number\_contig-100\_5.144066SRS142980\_contig\_number\_contig-100\_2561.36867SRS143722\_contig\_number\_contig-100\_34.89345SRS144753\_contig\_number\_525SRS147039\_contig\_number\_3835SRS147727\_contig\_number\_contig-100\_7883.7884SRS148424\_contig\_number\_28759SRS148721\_contig\_number\_55498SRS893383\_contig\_number\_contig-100\_4228.190527SRS971275\_contig\_number\_contig-100\_2969.275769
genus
129
816

1

SRS143523\_contig\_number\_14460
species
1739298


SRS045244\_contig\_number\_3331
species
1
674529

93975

SRS077335\_contig\_number\_3225SRS1041091\_contig\_number\_17502SRS1041138\_contig\_number\_10570
species
3

556259
1

SRS101433\_contig\_number\_12555
species

2292928
1

SRS143895\_contig\_number\_48683
species

817

SRS016897\_contig\_number\_416
species
1

820
70

SRS012849\_contig\_number\_26711SRS012969\_contig\_number\_contig-100\_314.241861SRS013098\_contig\_number\_42939SRS014287\_contig\_number\_contig-100\_57.120136SRS014459\_contig\_number\_40543SRS015065\_contig\_number\_8334SRS016056\_contig\_number\_5790SRS016954\_contig\_number\_26305SRS017247\_contig\_number\_2581SRS017701\_contig\_number\_contig-100\_819.132602SRS018575\_contig\_number\_8607SRS018936\_contig\_number\_2160SRS019397\_contig\_number\_8089SRS019445\_contig\_number\_15320SRS019808\_contig\_number\_5736SRS020328\_contig\_number\_contig-100\_1680.212268SRS020394\_contig\_number\_9422SRS021153\_contig\_number\_2226SRS022137\_contig\_number\_19784SRS022713\_contig\_number\_5408SRS023526\_contig\_number\_contig-100\_54.229707SRS024009\_contig\_number\_1498SRS024075\_contig\_number\_4758SRS024625\_contig\_number\_7573SRS045826\_contig\_number\_16661SRS048060\_contig\_number\_1582SRS049896\_contig\_number\_31789SRS049900\_contig\_number\_20096SRS051882\_contig\_number\_22616SRS054352\_contig\_number\_19136SRS055982\_contig\_number\_18203SRS056519\_contig\_number\_11589SRS057478\_contig\_number\_6239SRS058723\_contig\_number\_contig-100\_49.63284SRS062427\_contig\_number\_9911SRS064645\_contig\_number\_contig-100\_258.23328SRS074586\_contig\_number\_contig-100\_809.41582SRS075773\_contig\_number\_31931SRS076756\_contig\_number\_17332SRS076976\_contig\_number\_5493SRS077849\_contig\_number\_22301SRS098655\_contig\_number\_13908SRS098717\_contig\_number\_23186SRS100021\_contig\_number\_5028SRS1041036\_contig\_number\_4652SRS1041039\_contig\_number\_29651SRS1041116\_contig\_number\_8705SRS1041129\_contig\_number\_14714SRS1041137\_contig\_number\_30843SRS1041143\_contig\_number\_7194SRS1041144\_contig\_number\_2065SRS104327\_contig\_number\_6412SRS104400\_contig\_number\_13928SRS104975\_contig\_number\_24111SRS1054691\_contig\_number\_contig-100\_19.111148SRS1055043\_contig\_number\_24057SRS1055049\_contig\_number\_16163SRS1055067\_contig\_number\_16531SRS1055099\_contig\_number\_15479SRS142542\_contig\_number\_24717SRS143991\_contig\_number\_28594SRS144603\_contig\_number\_14051SRS147766\_contig\_number\_6382SRS148253\_contig\_number\_12664SRS148817\_contig\_number\_12399SRS893172\_contig\_number\_451SRS893187\_contig\_number\_737SRS893327\_contig\_number\_2400SRS893369\_contig\_number\_11478SRS971275\_contig\_number\_42996
species

1

SRS014313\_contig\_number\_4161
species
457395

585543
species

SRS011405\_contig\_number\_9571SRS048870\_contig\_number\_2960SRS053335\_contig\_number\_10655
3

4

SRS019030\_contig\_number\_15680SRS077454\_contig\_number\_13055SRS147977\_contig\_number\_7391SRS893342\_contig\_number\_4961
species
310298

246787
1
species

SRS016203\_contig\_number\_2066

28116
1

SRS050941\_contig\_number\_3925
species


SRS017859\_contig\_number\_2989SRS045645\_contig\_number\_5674SRS077552\_contig\_number\_720SRS893382\_contig\_number\_615
species
4
46506

2
species

SRS075984\_contig\_number\_24635SRS147919\_contig\_number\_16505
1946089

818
species

SRS147346\_contig\_number\_4926SRS148196\_contig\_number\_41196
2

291645
species

SRS1041134\_contig\_number\_457
1

1
species

SRS101376\_contig\_number\_1281
1263054

1239
1
phylum

class
1
186801

order
1
186802

family
1
186803

841
genus
1

166486
1
species

SRS054956\_contig\_number\_14542
